# ASSOCIATIONS OF NIGHT AND DAY SLEEP WITH COGNITIVE PERFORMANCE IN SHIFT WORKERS: PRELIMINARY RESULTS

**DOI:** 10.1093/geroni/igad104.1617

**Published:** 2023-12-21

**Authors:** Jinyoung Kim, Soonhyun Yook, Stephanie Nam, Hosung Kim, Mohammad Hesam Alavi, Joanna Oliver, Diane Lim

**Affiliations:** University of Nevada, Las Vegas, Las Vegas, Nevada, United States; University of Southern California, Los Angeles, California, United States; University of Nevada, Las Vegas, Las Vegas, Nevada, United States; University of Southern California, Los Angeles, California, United States; University of Nevada, Las Vegas, Las Vegas, Nevada, United States; University of Nevada, Las Vegas, Las Vegas, Nevada, United States; Miami VA Healthcare System, Miami, Florida, United States

## Abstract

Emerging evidence suggests that night shift workers are at higher risk of severe cognitive impairment, mainly because of the repeated disruption of the sleep–wake schedule and the resulting poor sleep (e.g., less deep sleep or shorter sleep time). However, questions remain about how daytime and nighttime sleep characteristics correlate with cognitive performance in shift workers. The purpose of this study is to examine the association of objective sleep quality, measured on workdays and days off, with cognitive performance, determined by the psychomotor vigilance task (PVT), in shift workers compared to day workers. We included eight night shift and ten day shift workers from an ongoing pilot study in this analysis. Participants undergo multiple home sleep studies with electroencephalograms on their workdays or days off. Sleep quality measures include total sleep time (TST), percentage of deep sleep stage in TST, and wake time after sleep onset (WASO). Night shift workers showed longer TST and shorter WASO during their nighttime sleep on days off compared to day shift workers. In night shift workers, longer deep sleep on days off was significantly correlated with faster mean reaction time to a visual stimulus (better cognitive performance) on the PVT (r = -.944, p = .016), after controlling for age. However, longer deep sleep during their daytime sleep was associated with slower reaction time (r = .756, p = .049). Further studies are necessary to elucidate differential impacts of daytime and nighttime sleep on the risk of future cognitive impairment in night shift workers.

